# The reality of every day communication for a deaf child using sign language in a developing country

**DOI:** 10.4314/ahs.v17i4.24

**Published:** 2017-12

**Authors:** Zandile M Blose, Lavanithum N Joseph

**Affiliations:** Discipline of Audiology, School of Health Sciences, University of KwaZulu-Natal, South Africa

**Keywords:** Deaf child, sign language, developing country

## Abstract

**Background:**

Research that focuses on the communication between deaf children and their hearing families is scarce despite the majority of deaf children being born into hearing families where a common communication mode needs to be forged.

**Objective:**

The aim of the study was to explore, describe and compare the nature of communication across typical daily contexts of a deaf child who uses South African Sign Language (SASL) and who is born into a hearing family with no prior experience of SASL.

**Methods:**

A case study design which included quantitative and qualitative components was used to observe a nine year old grade one child with profound hearing loss. Spontaneous communication was observed with 13 communication partners in the home context and these included the mother, a sibling and peers. Two educators and 11 peers were observed in the educational context. Surveillance cameras were used to obtain 27 hours of video-recording in the home and 19 hours at the school. Interviews were conducted with the mother, siblings, educators, and the deaf child.

**Results:**

It was observed that communication using SASL, albeit minimal, home signs, natural gestures and oral communication were used extensively. Due to a mismatch in the communication mode in the home context communication interactions were fewer and predominantly oral, impoverished and with frequent breakdowns whereas the communication interactions in the school were characterized by SASL, was meaningful and had fewer communication breakdowns.

**Conclusion:**

Communication for deaf children within the home is problematic as communication partners are not fluent in SASL.

## Introduction

The South African healthcare infrastructure is considered to be reasonable in terms of its development status when compared to other African countries. South Africa is considered to be a developing country. While there is paucity of research related to the prevalence of hearing loss in South Africa, Swanepoel et. al's^1^ report estimates of 3 in 1000 infants in the private sector are diagnosed with a congenital hearing loss compared to 4–6 infants in the public sector. The majority of deaf children are born into hearing families where a common communication mode does not exist as observed by Magnusson^2^. Yet research that focuses on the communicative interactions of deaf children and their hearing families is scarce, limited and outdated according to Klatter-Folmer et al^3^. Universally, deaf children are deprived of the ability to communicate and interact with people that they come into contact with both on a daily basis and less frequently. The lower case 'd’ in the word ‘deaf ‘ in a clinical context, is used to describe an individual's hearing status and refers to individuals with a profound hearing loss and who present with the inability to develop speech and language or benefit from speech reception as reported by Martin et al.^4^. On the other hand, Penn^5^ refers to the upper case ‘D’ in the term ‘Deaf’ as those individuals who have a hearing loss and share a common language and culture. Martin et al.^4^ further describes these Deaf individuals as part of the Deaf culture who use Sign Language and are considered a linguistic minority. The communication interactions between deaf and hearing individuals is a complex process as a common communication mode and understanding between the two communication partners is not readily available. Most^6^ claimed that communication is considered to be a social process and any interference with this process will have a considerable impact on interactions.

Language acquisition, whether signed or spoken is dependent on the hearing family members' ability to be responsive to their deaf child's communication needs and also to provide language that is visually and auditorily accessible as suggested by Volterra et al.^7^. According to Luckner et al.^8^ the hearing families of deaf children have limited understanding and knowledge of what it is like to be deaf. They fail to imagine a world in which speech is always either soft, simply not heard, distorted or unintelligible. What needs to be realized is that consistent twoway communication regardless of whether it is spoken or signed is essential. It is usually left to the family of the deaf child to select the communication option to be utilized; however, various factors need to be taken into consideration. Among these factors are the families' communication preference, the deaf child's communication needs and capabilities and the availability and accessibility of services. For Easterbrooks^9^ the communication options include manual communication, oral communication or a combination of both.

As deafness is viewed as a low-incidence disability, the majority of hearing families with a deaf child have never come into contact with a Deaf individual until the birth of their child or sibling, let alone, know Sign Language. Sign Language tends to exclude the hearing from the Deaf world, and the only way to communicate is either through writing or by Sign Language. Families of deaf children may also find this method of communication frustrating, since they will have to learn Sign Language in order to communicate with their children. This therefore raises the question of how deaf children who are enrolled in an educational setting where Sign Language is the primary method of communication interact and communicate with typical communication partners on a daily basis. How do communication interactions take place between hearing and Deaf individuals in a South African context that has to contend with issues of lack of early intervention, poor socio-economic challenges and limited facilities spread over vast geographical locations? There is limited research in this area. The study by Joseph et al.^10^ following parent-child interaction observations revealed inadequate communication between deaf children who use Sign Language and their parents in South Africa.

Knoors^11^ states that any parent, whether they have a deaf or hearing child is obliged to provide the child with education. Knoors^11^ further reports that deaf children may begin schooling from the age of three years to the age of twenty if they are enrolled in a special school. Deaf children born into hearing families continuously experience early and continued communication deprivation, family difficulties, inadequate educational experiences as well as social stigma and prejudice. Siegal^12^, suggested that “all Deaf and hard-of-hearing children deserve a quality, communication driven program, Language proficient teachers and staff who can communicate directly and at an adult level as a guiding and fundamental principle in the education sector for the Deaf”. Research regarding the deaf child's experience in a school for the Deaf with regards to classroom interactions with peers, playground interactions, and educator-child interactions is limited with little documentation of communication in this context.

Many studies have illustrated that deaf children's interactions with hearing peers are limited, as opposed to other Deaf peers according to Schoenwald-Oberbeck^13^. The deaf children's playmates are often obliged to respond to the deaf children by requesting for information, clarity or action during play. This is due to the communication breakdowns that occur during play. The interactions of deaf children with multiple peers are dependent on good language skills. Poor language skills limit the deaf child's interactions. The interaction of deaf children with their peers is an essential component in the development of social skills of these children. Communication interactions amongst peers also play a significant role in educational settings.

There is limited information in the area of communication interactions of Deaf children, who sign, especially in the South African context. The main concern for most families with deaf children is the practicality and functionality of communication in any context rather than the strict adherence to a particular communication method. According to Klatter-Folmer^3^, studies that investigated language development of deaf children who communicate using Sign Language within bilingual programmes is scarce and studies regarding Deaf verses hearing interaction partners are limited. This particular study aimed to investigate the communication interactions of a deaf child in two contexts, i.e. the home context and the school context, observing interaction with their family members, educators and peers. The purpose was to facilitate an understanding by hearing families and professionals who work with deaf children about the reality of the deaf child in terms of communication, interactions, from a South African perspective.

## Methods

**Figure 1:**
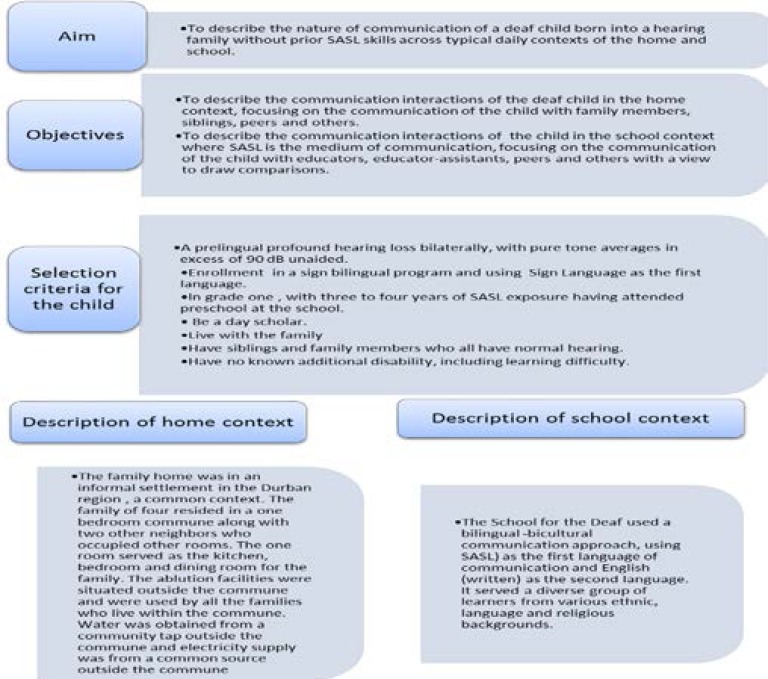
Representation of the aim, objectives, selection criteria and contexts of the study

A case study design which included quantitative and qualitative components was used to meet the aim of the study. Purposive sampling was used to select the participants of the study. Figure 1 below is representative of the aim and objectives of the study, selection criteria used and description of the home and school context.

**Figure 2:**
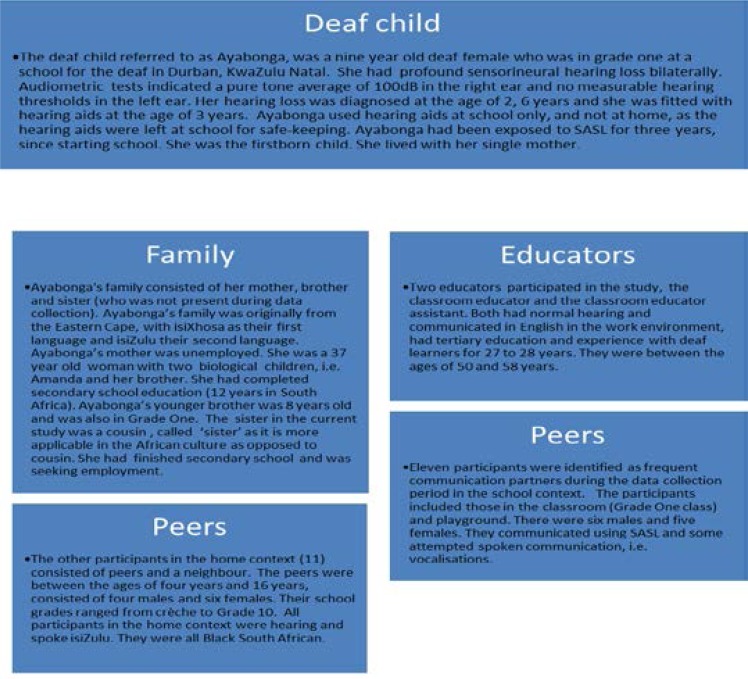
Description of participants in the home and school context

The participants of the study included the deaf child, family and hearing peers who were representative of the home context. The school context is represented by educators, educator assistants and Deaf peers. Figure 2 below represents a description of all the participants of the study.

**Figure 3:**
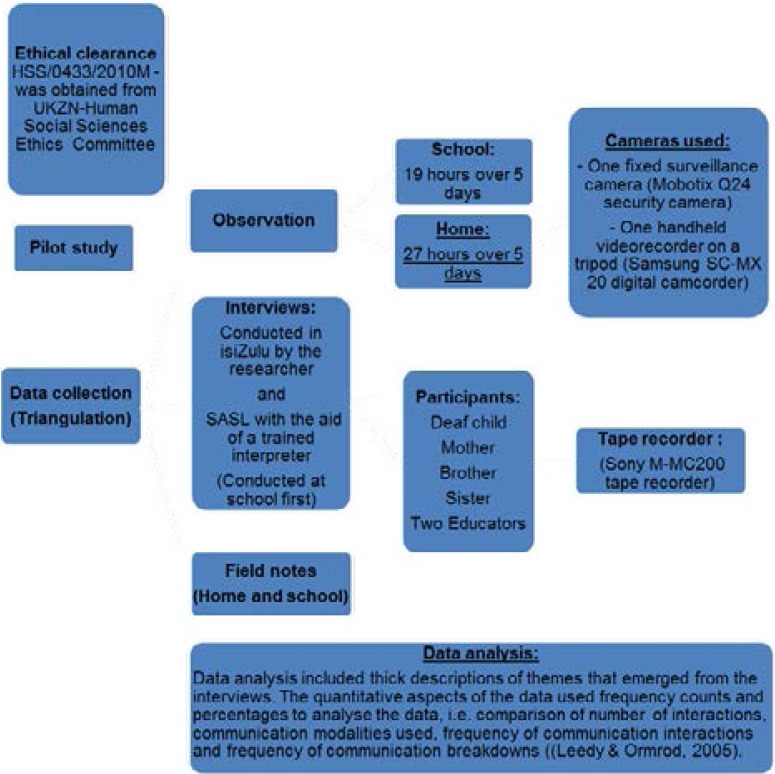
Data collection and analysis process

Spontaneous communication was observed with 13 communication partners in the home context and included the mother, a sibling and peers. Two educators and 11 peers were observed in the educational context. Surveillance cameras were used to obtain 27 hours of video-recording in the home and 19 hours at the school. Interviews were conducted with the mother, siblings, educators, and the deaf child. Figure 3 below is representative of the data collection and analysis process of the study.

## Results and discussion

### The nature of communication interactions

**Figure 4:**
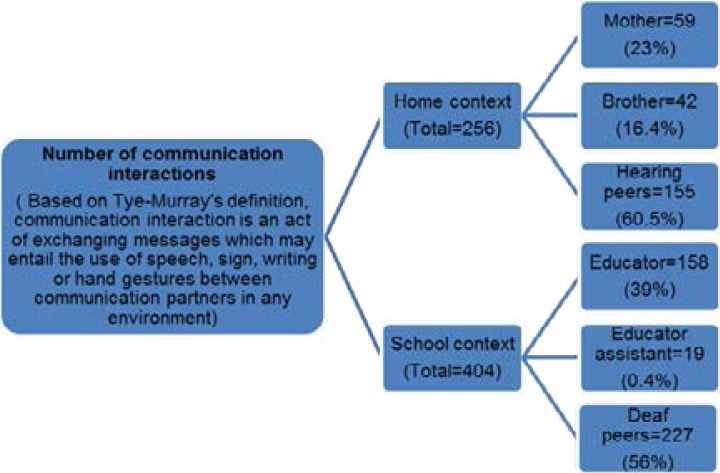
Number of communication interactions in the home and school context

Ayabonga's (not the real name of the participant) communication partners included family, educators and peers. Figure 4 represents the number of communication interactions between Ayabonga and the communication partners in the home and school context.

**Figure 5:**
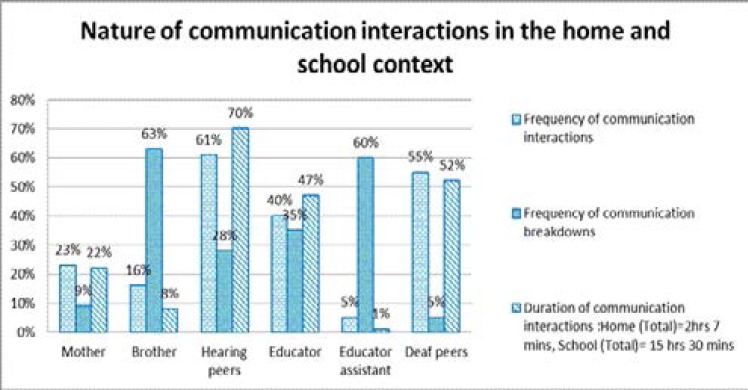
Nature of communication interactions

The peers in both contexts appeared to be the frequent communication partners. This may be attributed to the difference in the number of peers in both contexts compared to the family members and educators. Figure 5 below illustrates the nature of communication interactions in the home and school context in terms of the frequency of communication interactions, frequent communication partners, frequency of communication breakdowns as well as the duration of the communication interactions. It is evident that some form of communication interactions were taking place in both contexts, however the nature of these interactions in both contexts was different. It is evident from the home context that when communication interactions did take place, they were impoverished, limited in terms of duration and level of content within the interactions. These limitations had an impact on mother-child interactions, sibling interactions and to a certain extent on peer interactions. These findings were supported and contributed to the restrictions the mother reported in terms of parenting and teaching Ayabonga about life and social skills, morals and values, culture, boys, dating and sex as well as health risks such as HIV/AIDS, and pregnancy.

“*I long to teach her about boys, sex, HIV/Aids, but I don't know Sign Language well. She won't understand me, so I just keep quiet*”.

The quality of the communication interactions in the home raises concerns when compared to the communication interactions that took place in the school context. In the school context, Ayabonga was exposed to a linguistically rich environment which was quality driven in terms of duration and content as well as communication partners. The communication interactions in the school context seldom had communication breakdowns whereas in the home context, the communication breakdowns were common and evident. The differences in the communication interactions in both contexts raises concerns regarding the communication situation for Amanda. What are the implications of being exposed to two different environments daily in terms of one being communicatively rich and the other being communicatively impoverished?

### Modes of communication

**Figure 6:**
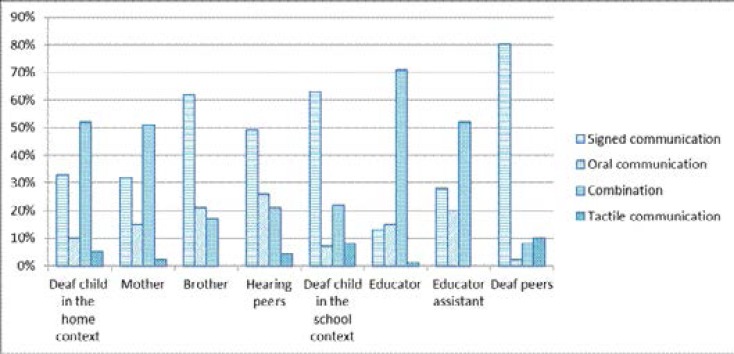
Communication modes used by all communication partners at home and school

The commonality of the communication modes used by Ayabonga and the different communication partners is a contributory factor to the ease and flow of communication interactions. Figure 6 illustrates the difference and frequency of modes of communication that Ayabonga used with communication partners in both contexts.

They used a variety of communication modes during interactions, and among these communication modes was oral communication in isiZulu and English depending on who the communication partner was, signed communication (pointing, gestures, home signs, eye gaze, facial expressions and SASL), a combination of all communication modes, and tactile communication (touch, pulling, etc). Results revealed that the majority of the participants used signed communication in the form of home signs, pointing and gestures frequently when communicating with Ayabonga. From the study it was evident that due to SASL not being a proficient language for the communication partners in the home context, the nature in which communication interactions took place was affected. In contrast to the home context, the communication partners in the school context were proficient in SASL thus the communication interactions were rich in nature. The commonality of a communication mode between communication partners, in this case, Amanda with hearing and deaf individuals, contributes to the level of closeness between herself and her communication partners. In the school, it was evident that there was a sense of belonging and no isolation of any form. The reality for Amanda and her family in the home context was a problematic communication environment. Due to SASL classes also not being accessible to the family, particularly the mother, options to improve communication interactions in such a context were futile. The mother only accessed some form of SASL classes from Amanda or when she was attending a school meeting where older deaf signing children were present. Considering that this arrangement was not happening on a daily basis, Amanda's mother was out of options. The family had to deal with deafness alongside other issues, e.g. ignorant neighbours, socio economic challenges, limited intervention options. Particularly for Amanda, she had to deal with multilingualism and had mastered the concept of code switching in both contexts. Depending on whom the communication partner was, Amanda could code-switch from SASL to home signs, or from English to isiZulu.

## Discussion

The purpose of the study was to describe and facilitate an understanding for hearing families and professionals who work with deaf children about the reality of living with a deaf child in terms of communication, interactions, schooling and language development from a South African perspective. The findings of the study revealed discrepancies in communication interactions between the child who is deaf and communication partners in different contexts, i.e. home and school. These differences in the communication interactions in both contexts raise concerns regarding the communication situation for Ayabonga and other children who are deaf who use Sign Language in similar contexts. What are the implications of being exposed to two different environments daily in terms of one being communicatively rich and the other being communicatively impoverished? From the study it was evident that due to communication partners in the home context not being proficient in SASL, communication interactions were not optimal. In contrast, the communication partners in the school context were proficient in SASL thus the communication interactions were rich in nature. The commonality of a communication mode between communication partners, in this case, Ayabonga with hearing and deaf individuals, contributes to the level of closeness between herself and her communication partners. In the school, it was evident that there was a sense of belonging and no isolation of any form. Professionals that are involved in the life of a child who is deaf need to stress the importance of having a common communication mode in the home context.

These professionals need to counsel families on the importance of selecting a communication mode and the implications of that choice. If the selected communication mode requires a new language to be acquired by the family, the audiologist needs to be able to provide clear guidelines about this in a supportive attitude and refer to relevant service providers who will be able to assist the family. This is critical as the importance of a common communication mode between the child who is deaf and hearing communication partners has been strongly illustrated in the current study. The reality for Ayabonga and her family in the home context was a problematic communication environment. Due to SASL classes also not being accessible to the family, particularly the mother, options to improve communication interactions in such a context were futile. The mother only accessed some form of SASL learning from Ayabonga or when she herself attended school meetings where older deaf signing children were present. Considering that SASL classes were not happening on a regular basis, Ayabonga's mother was out of options. The acquisition of a new language by hearing family members has financial and time implications also. This information needs to be provided to families during informational counselling. Audiologists need to place emphasis on the critical nature and importance of family centered intervention in a family where a child who is deaf exists regardless of the communication mode they are using. The family had to deal with deafness alongside other issues such as ignorant neighbours, socio economic challenges, and limited intervention options.

In the South African context, taking into consideration the low socio-economic background of most people, with high unemployment rates in the country, poverty and single mothers raising their children, one wonders about the feasibility of implementing family centered intervention. In many studies that have been conducted internationally as well as South African success stories, the benefits of family centered intervention are clearly illustrated, however, other factors that will contribute to failure on the implementation of family centered intervention need to be considered. For the current study, Ayabonga's mom was a single parent who was unemployed. Prior to any potential collaboration with the professionals, she had to think of the family's basic needs and prioritize which outweighed the other in terms of importance for quality of life for the whole family. Roush et al.14 concluded that empirical research regarding the implementation of family centered intervention is lacking. In the study by Calderon et al.15 28 hearing families with children who are deaf in the United States between the ages 42 and 87 months were interviewed. The study also included children who are deaf who were aged between 9 to 42 months postgraduation from an early intervention program.

The researchers took the following factors into consideration: demographics, duration and intensity of early intervention, parent involvement, educational and communication choices. Since the study considered children from rural areas, various obstacles were anticipated. Among these obstacles was less accessibility to intervention programs, less parent to parent support, higher rates of unemployment, fewer professional resources, financial stress, less accessibility to deaf adult role models and less developed educational programs. These factors may have an impact on early intervention. The children were seen three times per month. The results revealed that the language scores clearly outlined that the intervention was not adequate to compensate for early auditory and language deprivation. These results revealed that parents needed to develop fluency in the communication modes adopted and provide one or two hour “instruction” per week. The gaps identified by researchers and the obstacles seen in the implementation of early intervention programs are similar to this South African study. Ayabonga was from an informal settlement context in which the above mentioned obstacles regarding the implementation of early intervention were applicable. Audiologists in South Africa together with various stakeholders need to offer early intervention programs which are family orientated while taking into consideration possible obstacles.

## Strengths of this study

The gathering of data via multiple methods increased reliability and validity of the study as the researcher was able to triangulate the data. The child who was deaf was observed in natural settings. The researcher was a first language isiZulu speaker who the family and community were able to identify with linguistically and culturally during the observations in the home context and this enhanced the quality of the data collection. As this was a single case study, it allowed the researcher to obtain indepth and detailed data. The information obtained from the participants through interviews and observations was valuable to create an understanding of how communication interactions take place between a deaf signing child in a home and school context in South Africa. The study highlighted the multimodal nature of communication and challenges experienced by a child who uses Sign Language with hearing communication partners.

## Limitations of this study

A detailed analysis of the language components were not fully explored in terms of content, form and use for both signed and oral communication. Similarly, the components of signed communication in terms of use of SASL, home signs, pointing and showing, and pantomime were not individually analyzed and compared. The signing proficiency of participants in the home context was not explored fully to measure communication partners SASL skills and vocabulary.

## Conclusion

The research was motivated by the need to investigate how communication interactions take place in the home with hearing individuals following the acquisition of Sign Language by the child who is deaf. The school context was used as a reference to compare the nature of communication interactions in these two contexts. The findings of the study revealed that the child who is deaf presented with metalinguistic skills in that she used differing communication modalities to accommodate the different communication partners and was able to understand each communication partner in her environment. Idiosyncratic home signs were still evident in the home due to little if any knowledge of SASL by the deaf child's communication partners. The home signs allowed for communication interactions to take place so that the child not isolated, however, the quality and type of communication interactions were of concern. The communication interactions were limited and impoverished due to a common communication mode not being readily available between the child who is deaf and hearing participants in the home context. The lack of mode matching appeared to be a core contributing factor to the limited communication interactions in the home and the increased communication breakdowns. On the other hand, contrasting results were obtained in the school context. Meaningful communication interactions were reported and observed in the school context. Minimal communication breakdowns were observed in this context compared to the home context. The importance of the role that needs to be played by the audiologist was clearly evident as it has implications for the communicative development of the child who is deaf. The importance of detailed and appropriate informational counseling, facilitation of family centered intervention and counseling and the implementation of early intervention programs were highlighted. The study highlighted the importance of Sign Language acquisition by hearing communication partners to assist in improving communication interactions with individuals who are deaf, particularly family members. From the study, it was evident that despite the availability of policies related to deaf education and implementation of intervention services by audiologists, there appears to be limited or no evidence on the practical implementation of such policies. This places an unfair burden on the child who has to take responsibility for establishing and maintaining communication interaction particularly in the home environment.
